# Lack of activity of HIV-1 integrase strand-transfer inhibitors on recombinase activating gene (RAG) activity at clinically relevant concentrations

**DOI:** 10.1128/spectrum.02468-24

**Published:** 2024-11-19

**Authors:** Sally Demirdjian, Vincent N. Duong, Jennifer N. Byrum, Arabinda Nayak, Cooper B. McKinney, Jason K. Perry, Christian Callebaut, Karla K. Rodgers, Brie Falkard, Joy Y. Feng

**Affiliations:** 1 HIV Clinical Virology, Gilead Sciences, Inc., Foster, California, USA; 2 Discovery Sciences and Technologies, Gilead Sciences Inc., Foster, California, USA; 3 Department of Microbiology and Immunology, University of Oklahoma Health Sciences Center, Oklahoma, Oklahoma, USA; 4 Protein Therapeutics, Gilead Sciences Inc., Foster, California, USA; 5 Structural Biology and Chemistry, Gilead Sciences Inc., Foster, California, USA; Kumamoto Daigaku, Kumamoto, Kumamoto, Japan

**Keywords:** HIV-1, recombinase activating gene, INSTI, integrase, V(D)J recombination, RAG1/RAG2

## Abstract

Human immunodeficiency virus 1 (HIV-1) infection remains a global health concern, with nearly 30 million people on antiretroviral (ARV) treatment. Integrase strand-transfer inhibitors (INSTIs) that block HIV-1 integrase are crucial components of first-line combination ARV therapies recommended in most international guidelines and have significantly improved HIV-1 treatment due to their efficacy and safety. This study evaluates potential off-target effects of INSTIs on recombinase activating genes (RAG1 and RAG2), which are essential for adaptive immune system function. We performed a comprehensive assessment of the off-target effects of clinically approved INSTIs on RAG activity, utilizing both biochemical and cellular assays. We purified the first catalytically active recombinant human core RAG1–RAG2 complex and tested it in the presence of the co-factor human HMGB1 protein for the gel-based biochemical RAG DNA cleavage assay. Additionally, we optimized an extrachromosomal V(D)J recombination cellular assay using murine mCherry-core RAG1, full-length murine mCherry-RAG2, and a plasmid substrate green fluorescent protein (GFP) reporter system, transfecting them into cells in the absence or presence of inhibitors. This setup enabled high-throughput analysis of V(D)J recombination for multiple compounds in a dose–response manner via flow cytometry. Physiologically relevant concentrations of INSTIs were examined for their potential impact on RAG activity and V(D)J recombination, with approved INSTIs showing minimal to no effects on recombinase activity. Consequently, the findings support the continued use of INSTIs in HIV-1 treatment without substantial concern for adverse effects on V(D)J recombination and immune system function.

**IMPORTANCE:**

INSTIs are a crucial component of antiretroviral treatments for HIV-1 infection. This study provides a careful and thorough analysis of the impact of approved INSTIs on recombinase activating gene (RAG1 and RAG2) activity, which plays a pivotal role in the adaptive immune system. The concentrations tested were derived from several clinical studies and accounted for the maximum free fraction of the drug available in patients. This approach ensures that our findings are directly applicable to clinical scenarios by providing meaningful insights into the potential drug side effects in patients. We developed biochemical and cellular assays to measure the impact of INSTIs on RAG activity. All tested INSTIs did not inhibit RAG at supratherapeutic concentrations in the RAG1/RAG2 biochemical cleavage and cellular V(D)J recombination assays. Our assessment supports the continued use of INSTIs in HIV-1 treatments without concern for adverse effects.

## INTRODUCTION

Human immunodeficiency virus 1 (HIV-1) infection remains a serious and life-threatening disease of major public health significance, with approximately 39 million people with HIV-1 (PWH) worldwide and an estimated 29.8 million on antiretroviral (ARV) treatment (1). Standard of care for the treatment of HIV-1 infection involves the use of a combination of ARV drugs to suppress viral replication to below detectable limits, increase CD4 cell counts, and delay disease progression. HIV-1 treatment guidelines recommend integrase strand-transfer inhibitor (INSTI)-based regimens for both initial and switch antiretroviral therapies (ARTs) in PWH (U.S. Department of Health and Human Services, European AIDS Clinical Society, and International AIDS Society guidelines) and have played an ever-growing role in the treatment of HIV-1 due to their proven efficacy and safety (2). INSTIs block the strand-transfer step of HIV-1 DNA integration into the host genome to inhibit HIV-1 replication. These drugs bind the active site of the catalytic core domain of HIV-1 integrase, making several molecular interactions with the viral DNA and integrase protein (3, 4). More specifically, INSTIs work by binding to magnesium metal cations at the integrase active site within the intasome (integrase–viral DNA complex), which disrupts the 3′ terminal nucleotide of the viral DNA and prevents the formation of a covalent bond between the viral DNA and the host DNA. This inhibition effectively stops the viral DNA from being incorporated into the host genome.

There are currently five INSTIs approved for the treatment of HIV-1 infection: raltegravir (RAL), elvitegravir (EVG), dolutegravir (DTG), bictegravir (BIC), and cabotegravir (CAB). RAL, the only INSTI that is not part of a fixed drug combination, was approved for once- and twice-daily oral dosing. EVG was approved as part of two regimens in combination with either emtricitabine (FTC)/tenofovir disoproxil fumarate or FTC/tenofovir alafenamide. DTG and BIC were later developed and approved for use in daily oral regimens, while CAB is used as a part of a long-acting injectable regimen. The recently approved INSTIs (DTG, BIC, and CAB) have significantly improved resistance profiles, retaining potency against many of the mutants selected by the earlier approved INSTIs, RAL and EVG (2). CAB, a potent analog of DTG, is the first available INSTI delivered by a long-acting injection, administered intramuscularly either once monthly or every 2 months in combination with the non-nucleoside reverse transcriptase inhibitor rilpivirine. RAL, EVG, DTG, and BIC are approved for use in ART-naïve and treatment-experienced PWH, while CAB-RPV is approved to replace a stable oral regimen in patients with viral suppression. Long-acting injectable cabotegravir is also approved for pre-exposure prophylaxis (5, 6).

It was previously reported (7–9) that HIV-1 integrase inhibitors can potentially inhibit the cellular recombinase activating genes (RAG1 and RAG2). RAG proteins play a pivotal role in the adaptive immune system by initiating the process of V(D)J recombination to generate diverse antigen receptors such as T-cell receptors and immunoglobulins. This controlled rearrangement of genetic segments by the RAG machinery ensures a diverse immune system repertoire for recognizing and fighting a large variety of pathogens. Consequently, inhibition of RAG and, subsequently, T- and B-lymphocyte receptor formation, prevents maturation and development of T and B cells, resulting in immunodeficiencies (10, 11). Biochemically, RAG1 and RAG2 are DNA recombinases that recognize recombination signal sequences (RSSs), which are defined by conserved heptamers or nonamers with spacers of either 12 or 23 base pairs (bp) (12RSS/23RSS) (12, 13). At these sites, RAG1 and RAG2 produce double-strand DNA breaks to initiate recombination. RAG1 primarily plays a catalytic role, while RAG2 functions as a critical accessory subunit by enhancing recognition of RSS substrates (14). Additionally, high-mobility group box proteins (HMGB1) behave as co-factors and promote RAG1/RAG2 binding and cleavage at these RSS sites by bending the DNA substrate. This molecular bending promotes RAG forming a complex with one 12RSS and one 23RSS (15, 16). The catalytic mechanism of RAG proteins is similar to that of transposases and retroviral integrases and shares structural features like the RNase H-like fold, where their catalytic triads reside (aspartate–aspartate–glutamate [DDE] motif) (7, 17–20). With these similarities in mind, it was hypothesized that HIV-1 integrase inhibitors may cause off-target inhibition of RAG1/RAG2 (7).

Early studies showed that compounds in the initial diketo acid class (p8 and p10), which have integrase inhibitory activity, inhibited RAG1/RAG2 *in vitro* by interfering with binding of DNA with RSS at high concentrations (p8 IC_50_ = 200 µM and p10 IC_50_ = 20 µM) (7). Subsequent studies assessed if HIV-1 integrase inhibitors, EVG, DTG, and RAL, could have off-target effects on RAG1/RAG2 using polyacrylamide gel electrophoresis (PAGE) binding and cleavage assays (8, 9). EVG was reported to bind to the central domain of RAG1 to mediate inhibition of RAG cleavage at the RSS at concentrations tested (50–200 μM), with no effect on binding or cleavage being observed with RAL up to the highest concentration tested (200 µM) (9). Follow-up experiments using DTG (200–500 μM) showed that DNA binding and cleavage by RAG1 decreased approximately 50%–75% at DTG concentrations tested, demonstrating weaker inhibition of RAG by DTG compared to EVG (8). However, in these *in vitro* studies, the concentrations of EVG and DTG required to inhibit RAG1 cleavage activity greatly exceeded (several magnitudes higher) those observed in HIV-1-infected individuals when treated with these drugs. As integrase inhibitors are highly protein bound, it is important to consider the maximum plasma concentration and the free drug fraction that is relevant in clinical concentrations (0.70% for DTG, 0.48% for EVG, and 12.18% for RAL) (21–23). Given the non-physiological concentrations previously tested *in vitro*, assessment of potential off-target effects of INSTIs on RAG activity using clinically relevant concentrations is a current gap in the literature (8, 9).

In this study, we assessed a set of approved HIV-1 integrase inhibitors for their potential to inhibit RAG1/RAG2 in two novel testing systems: human recombinant proteins RAG1/RAG2/HMGB1 in biochemical assays and murine extrachromosomal cell-based V(D)J recombination assays. We demonstrate that at clinically relevant concentrations of these inhibitors, the previously reported off-target effects on recombinase activity are not found in this study. These findings affirm the ongoing utilization of INSTIs in HIV-1 treatment.

## RESULTS

### Purification of human RAG1/RAG2 and HMGB1 proteins

**Fig 1:**
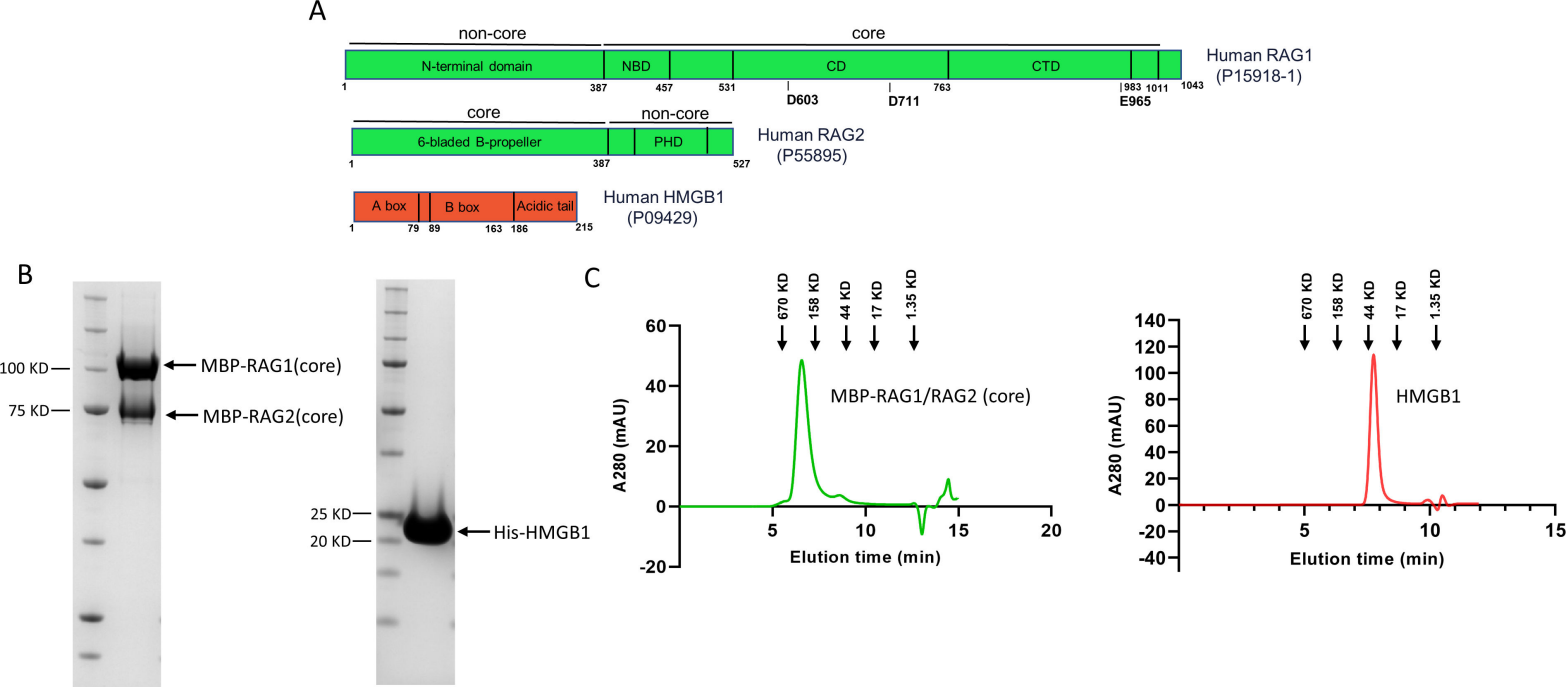
Purification of RAG1/RAG2 and HMGB1 proteins. (**A**) The human RAG1 protein (UniProt ID P15918-1) is 1,043 amino acids long and consists of a N-terminal domain (AA 1–386) and core domain (AA 387–1,011). The core domain contains a nonamer DNA-binding domain (NBD) (AA 387–457), a central domain (CD) (AA 531–763), and a C-terminal domain (CTD) (AA 764–983). Active site motif Asp–Asp–Glu (D603, D711, and E965) on RAG1 is indicated. The RAG2 protein (UniProt ID P55895) is 527 amino acids long and contains a N-terminal core domain (AA 1–387) that folds into a six-bladed b-propeller domain and a C-terminal plant homeodomain (PHD). The HMGB1 protein (UniProt ID P09429) is 215 amino acids long and is organized into two homologous DNA-binding domains, A-box (AA 1–79) and B-box (AA 89–162) and a C-terminal acidic tail (AA 186–215). (**B**) SDS-PAGE of the purified RAG1/RAG2 and HMGB1 proteins. (**C**) SEC–HPLC analysis of RAG1/RAG2 and HMGB1 proteins. Arrows indicate the elution point of molecular weight standards (chromatogram not shown).

The schematic of full-length (FL) human RAG1 and RAG2 proteins, including the active site residues (DDE motif) in RAG1 and catalytically essential core regions of each protein, are shown in Fig. 1A. We successfully expressed the human core RAG1–core RAG2 complex by transfecting plasmids encoding core domains (instead of the FL versions) into human cells. The core regions of RAG1 and RAG2 are sufficient to catalyze V(D)J recombination, whereas the non-core regions regulate the recombination reaction (13). We also expressed the recombinant HMGB1 protein by transforming the plasmid encoding N-terminal DNA-binding domains into *Escherichia coli* BL21DE3 cells (Fig. 1A). The sodium dodecyl–sulfate PAGE of the purified RAG1/RAG2 and HMGB1 proteins confirmed their expected sizes (Fig. 1B). Analytical size exclusion chromatography–high-performance liquid chromatography (SEC–HPLC) quality control analysis was performed using a Superdex 200 Increase 5/150 GL column, which indicated the heterotetrametric status of the purified RAG1/RAG2 proteins (~ 400 kD) (Fig. 1C). For purified HMGB1 protein, the analytical SEC–HPLC data suggest the protein is dimerized (monomer size ~18.9 kD) from the elution pattern observed (Fig. 1C). However, further confirmatory analysis by size exclusion chromatography–multi-angle light scattering indicated that the molecular weight (MW) of the HMGB1 protein was consistent with the approximate monomeric size of the protein, as expected (Fig. S1). Intact liquid chromatography–mass spectrometry (LC–MS) analysis was used to verify the MW and identity of RAG1/RAG2 and HMGB1 proteins (Fig. S1). The *A*_260_/*A*_280_ of the final protein preparations was calculated to be 0.58–0.59 using a NanoDrop spectrophotometer, suggesting negligible nucleic acid contaminations. It should be noted that the human RAG1/RAG2 core protein preparation retained maltose-binding protein (MBP) tags, as described in Materials and Methods.

### INSTIs do not inhibit human RAG1/RAG2 at clinical concentrations in biochemical assay

**Fig 2:**
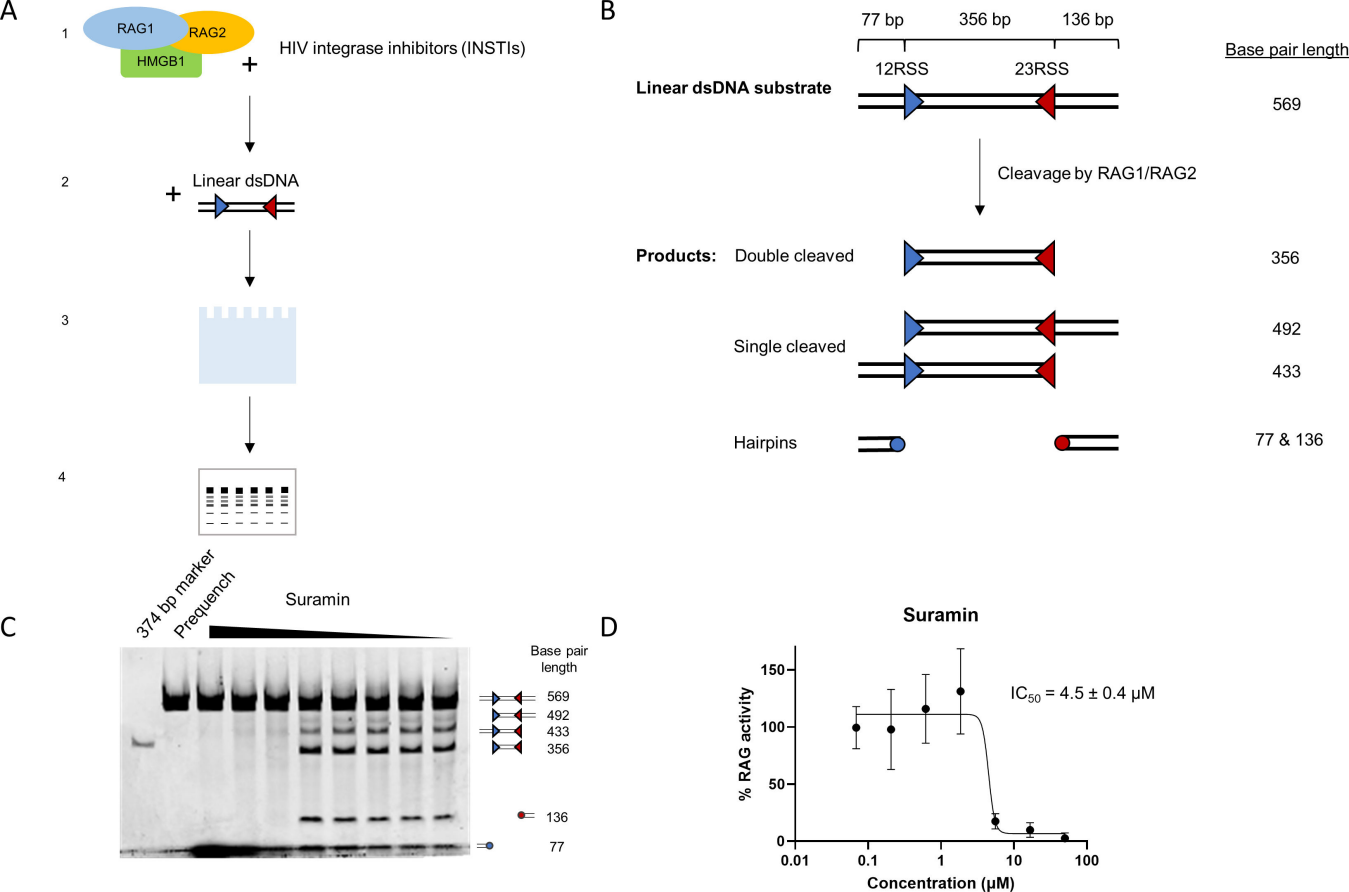
Design of biochemical assay to assess recombinant RAG1/RAG2 cleavage activity. (**A**) Experimental workflow of biochemical assay. (1) Co-expressed RAG1 and RAG2 are pre-incubated with HMGB1 in the presence of INSTIs, (2) the reactions are initiated upon addition of dsDNA containing 12 and 23RSS, (3) reactions are quenched and resolved via native gel electrophoresis, (4) gels are stained with SYBR Gold and substrate and products are quantified. (**B**) The 569-bp linear dsDNA substrate was designed to contain a 12RSS and 23RSS that is cleaved by RAG1/RAG2 under experimental conditions. The substrate was designed so that the central fragment after successful cleavage of both RSSs produces a 356-bp dsDNA and 77- and 136-bp fragments that become a hairpin. (**C**) Suramin was used as a tool compound to inhibit RAG1/RAG2 activity. As a control, both a 374-bp marker and a pre-quenched reaction were loaded alongside the samples. (**D**) % RAG activity was plotted for suramin. The IC_50_ of suramin was calculated to be 4.5 µM.

To directly observe the potential inhibition of RAG1/RAG2, we measured the effect of INSTIs on DNA cleavage by RAG1/RAG2 in the presence of HMGB1. This biochemical system utilizes pre-assembled recombinant human core RAG1 (AA387-1011)/core RAG2 (AA1-387) in the presence of excess truncated HMGB1 (AA1-163) and a linear dsDNA RSS substrate, using gel electrophoresis and a fluorescent dye to monitor cleavage products (Fig. 2A) (24). The dsDNA RSS substrate, containing both the 12RSS and 23RSS, was designed to have the different cleavage products resolved via gel electrophoresis. RAG1/RAG2 can singly cleave either the 12RSS or the 23RSS, or both, leading to potentially five different products in the reaction (Fig. 2B). HMGB1 orients RAG1/RAG2 for proper binding to the RSS substrate (16). We quantified the activity of RAG as the percentage of substrate conversion to a specific cleaved product, as separated by agarose gel electrophoresis and determined by the DNA-binding fluorescent dye. With the 569-bp substrate, a single cleavage of the 12RSS results in a 492-bp dsDNA and a 77-bp hairpin. Cleavage of the 23RSS alone produces a 433-bp dsDNA and a 136-bp hairpin. Successful cleavage of both RSSs results in a 356-bp dsDNA and both of the 77- and 136-bp hairpins. For the RAG activity analysis, we measured the amount of double cleaved product (356 bp) in relation to the amount of substrate (569 bp) at the end of the reaction. The optimal concentrations of RAG1/RAG2, HMGB1, and MgCl_2_ were determined through titration (Fig. S2). Because there is no direct RAG1/RAG2 inhibitor previously characterized, we used suramin, a polyanionic compound commonly used as a non-specific inhibitor for DNA/RNA binding or processing proteins as a control for inhibition (25, 26). This compound showed an IC_50_ of 4.5 µM in the biochemical RAG assay (Fig. 2C and D).

**Fig 3:**
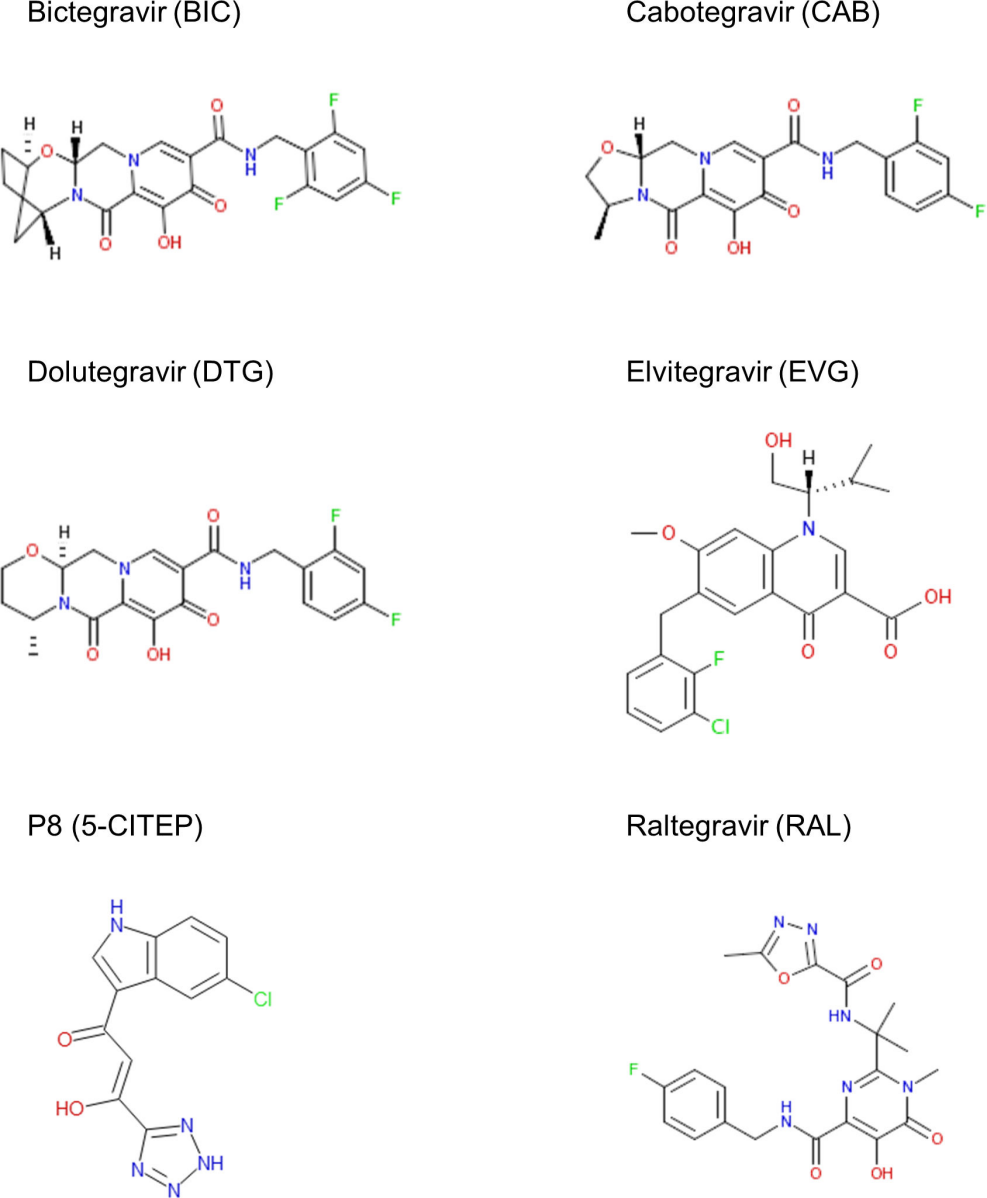
Chemical structures of integrase inhibitors. The chemical structures of bictegravir (BIC), cabotegravir (CAB), dolutegravir (DTG), elvitegravir (EVG), p8 5-CITEP, and raltegravir (RAL) are shown. Nitrogen (N), oxygen (O), and fluorine (F) or chlorine (Cl) are labeled in blue, red, and green, respectively.

Using this multi-protein system, we tested six HIV-1 integrase inhibitors (Fig. 3) at starting concentrations that are clinically relevant, taking into consideration the free *C*_max_ of these inhibitors (Table 1). Free *C*_max_ was calculated based on the reported human clinical maximum plasma concentrations observed in clinical trials and accounting for protein binding of integrase inhibitors by serum proteins (21–23, 27–29) (Table 1). Notably, our biochemical system does not include a serum component. Therefore, the free compound concentrations are supratherapeutic and expected to be significantly greater than the free concentration of these compounds in patients (~5.3- to 26-fold higher than free *C*_max_). We included among these inhibitors, p8 (5-CITEP), one of the prototype integrase inhibitors that was found to inhibit murine RAG1/RAG2 in a previous study as an inhibitor control in our experiments (7).

**TABLE 1:** INSTI dose selection and IC_50_ in biochemical RAG assay^*e*^

Drug	Clinical dosage^*c*^	Human clinical *C*_max_ (µM)^*c*^	% Free^*c*^	Human clinical free *C*_max_ (µM)^*c*^	Starting dose selected for dilution (µM)	IC_50_ biochemical assay (µM)	Target IC_50_ for integrase (nM)^*d*^
BIC	50 mg	13.7	0.25	0.034	0.5	>0.5	7.5 ± 0.3
CAB^*a*^	IM 400 mg PO 30 mg	15.7	0.16	0.025	0.5	>0.5	3
DTG	50 mg	8.3	0.70	0.058	0.5	>0.5	7.4 ± 0.6
EVG^*b*^	150 mg	4.7	0.48	0.022	0.5	>0.5	8.4 ± 0.7
RAL	1,200 mg	15.7	12.18	1.900	10	>10	3.3 ± 0.6
p8	N/A	N/A	N/A	N/A	400	58.2 ± 5.6	~200,000
Suramin	N/A	N/A	N/A	N/A	50	4.5 ± 0.4	N/A

^*a*^ Long-Acting Antiretroviral Treatment Enabling Trial (LATTE).

^*b*^ Administered with 150 mg cobicistat.

^*c*^ Values obtained from reference (29).

^*d*^ Binding to recombinant HIV-1 integrase/strand-transfer assays. Data for BIC, DTG, and EVG are from reference (30). Data for CAB and RAL are from references (31, 32), respectively, and data for p8 are from reference (7).

^*e*^ BIC, bictegravir; CAB, cabotegravir; *C*_max_, clinial maximum plasma concentration; DTG, dolutegravir; EVG, elvitegravir; IC_50_, inhibitory concentration giving 50% inhibition of enzymatic activity; INSTI, integrase strand-transfer inhibitor; N/A, not available; RAL, raltegravir.

**Fig 4:**
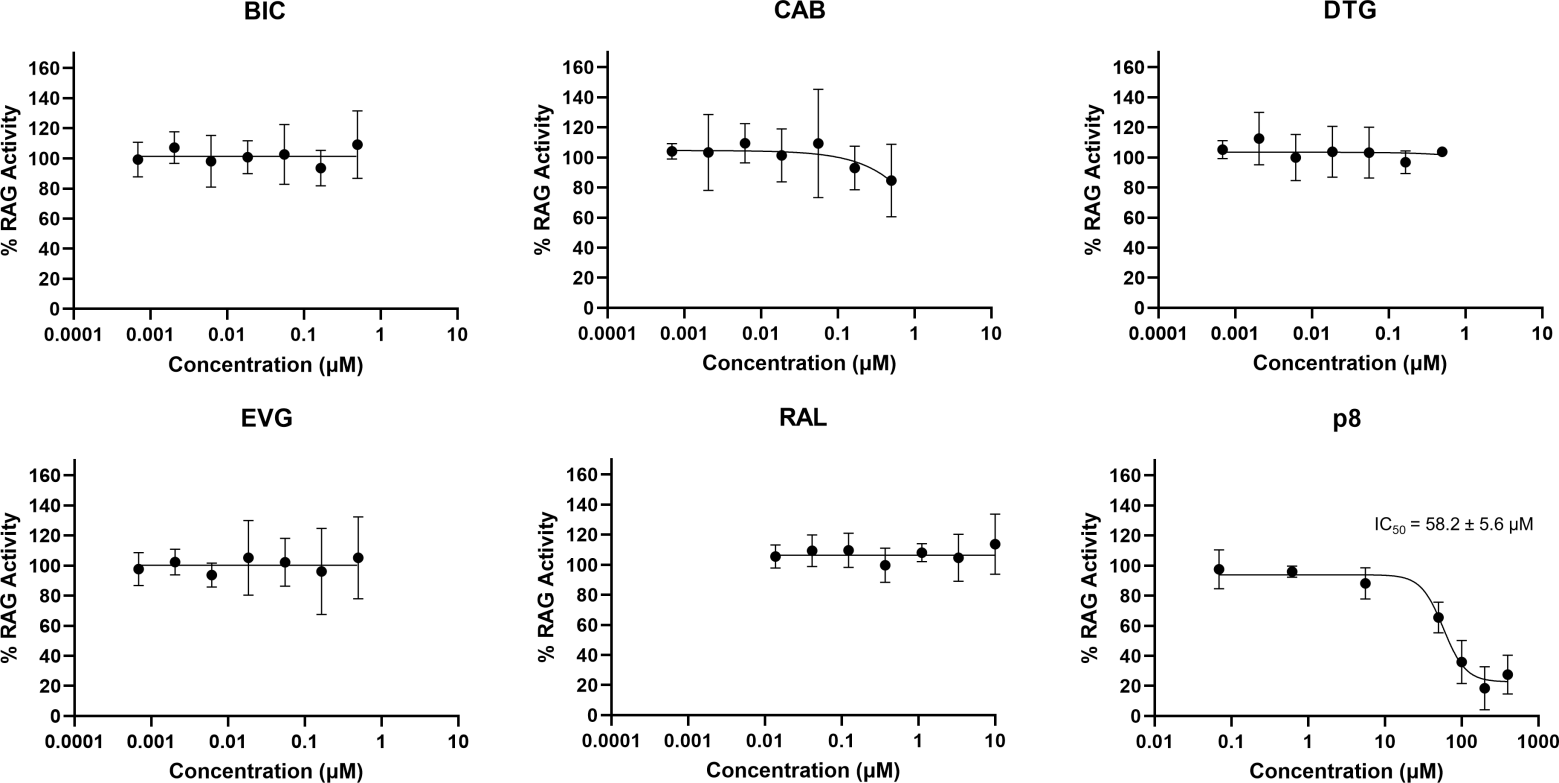
Biochemical inhibition of RAG1/RAG2 cleavage by INSTIs. Co-expressed RAG1 and RAG2 were pre-incubated with HMGB1 in the presence of INSTIs, with reaction initiation upon addition of dsDNA containing 12RSS and 23RSS. The densities of the 569-bp-length substrate and fully cleaved 356-bp-length product were quantified, after resolving via native gel electrophoresis. The product density was divided by the sum of substrate and product bands to obtain a percent product value. These calculated values were subtracted from a negative control (pre-quenched reaction where EDTA is added before the reaction is initiated) and then normalized to the dimethyl sulfoxide-treated control. CAB, DTG, RAL, EVG, and BIC do not show inhibitory activity against RAG1/RAG2 at clinically relevant concentrations. p8 displayed inhibitory activity against RAG1/RAG2 with an IC_50_ of 58.2 µM.

Upon testing the HIV-1 integrase inhibitors in our biochemical RAG assay, we observed that the five approved INSTIs did not inhibit RAG at supratherapeutic concentrations (BIC, CAB, DTG, EVG IC_50_ >0.5 µM; RAL IC_50_ >10 µM) (Fig. 4). We were able to show that p8 inhibits RAG (IC_50_ = 58.2 ± 5.6 µM) (Fig. 4), corroborating the previous findings from the literature (7). These biochemical results showed no indication of off-target effects on RAG1/RAG2/HMGB1-catalyzed DNA processing under these experimental conditions at clinical concentrations of these integrase inhibitors.

### Extrachromosomal V(D)J recombination assay and plasmid design

**Fig 5:**
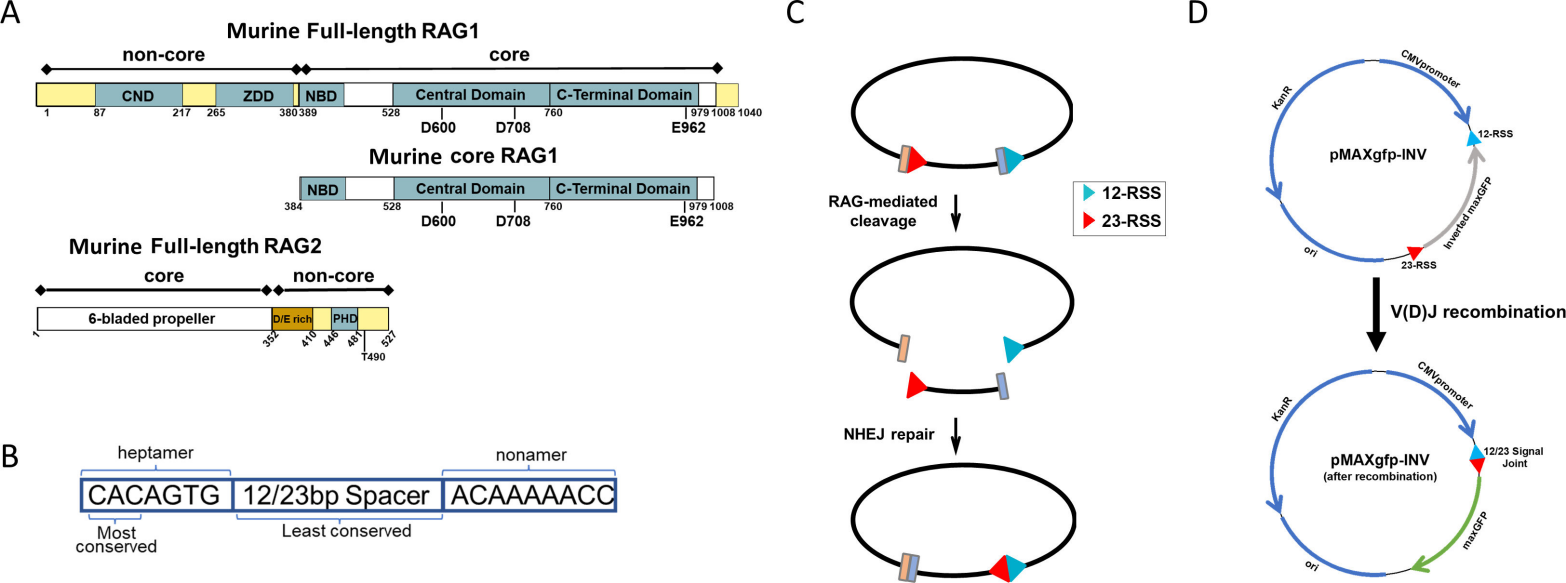
Design of cellular assay to assess RAG1/RAG2 recombinase activity. (**A**) Schematic of FL mouse RAG1 and RAG2 proteins. The active site residues (the DDE motif) in RAG1, as well as the core regions of each protein, are indicated. (**B**) The consensus RSS contains a conserved heptamer and nonamer separated by 12 or 23 non-conserved bp (12RSS and 23RSS). (**C**) The extrachromosomal V(D)J recombination assay. (**D**) pMAXgfp-INV before and after V(D)J recombination.

To further evaluate the effect of HIV-1 integrase inhibitors on RAG activity in a cellular context, we optimized a novel extrachromosomal cell-based V(D)J recombination assay, enabling assessment of multiple compounds in a dose–response manner by flow cytometry (33). The schematic of FL murine RAG1 and RAG2 proteins, including the active site residues (DDE motif) in RAG1 and catalytically essential core regions of each protein, are shown in Fig. 5A. The cell-based assay used vectors that encode for the core RAG1 protein (instead of the FL version) and FL murine RAG2 expressed as C-terminal fusions to mCherry, termed mCherry-core RAG1 and mCherry-RAG2. The core regions of each RAG protein are sufficient to catalyze V(D)J recombination, whereas the non-core regions function to regulate the recombination reaction (13). Moreover, FL RAG1 tends to express at significantly lower levels with enriched localization to nucleoli, leading to lower activity on the extrachromosomal plasmid substrates (34) (J. N. Byrum and K.K. Rodgers, unpublished data). The consensus RSS used in this assay contains a conserved heptamer and nonamer separated by 12 or 23 non-conserved bp (12RSS and 23RSS) (Fig. 5B). A general schematic of the design for the plasmid substrate with the 12RSS and 23RSS orientations before and after V(D)J recombination is presented in Fig. 5C. More specifically, the plasmid substrate pMAXgfp-INV was designed with a 12RSS and a 23RSS flanking each side of the *Pontellina plumata* green fluorescent protein gene (termed maxGFP), where the gene is in an inverted orientation relative to the CMV promoter (Fig. 5D). The RSSs are positioned in a co-linear orientation, such that successful V(D)J recombination activity will result in inversion of the maxGFP gene to place it in a transcriptionally active orientation with resulting expression of GFP. Detection of GFP-expressing cells using microscopy and flow cytometry was used as a direct readout of V(D)J recombination activity.

### Microscopy and PCR sequencing validation of cell-based V(D)J recombination assay

**Fig 6:**
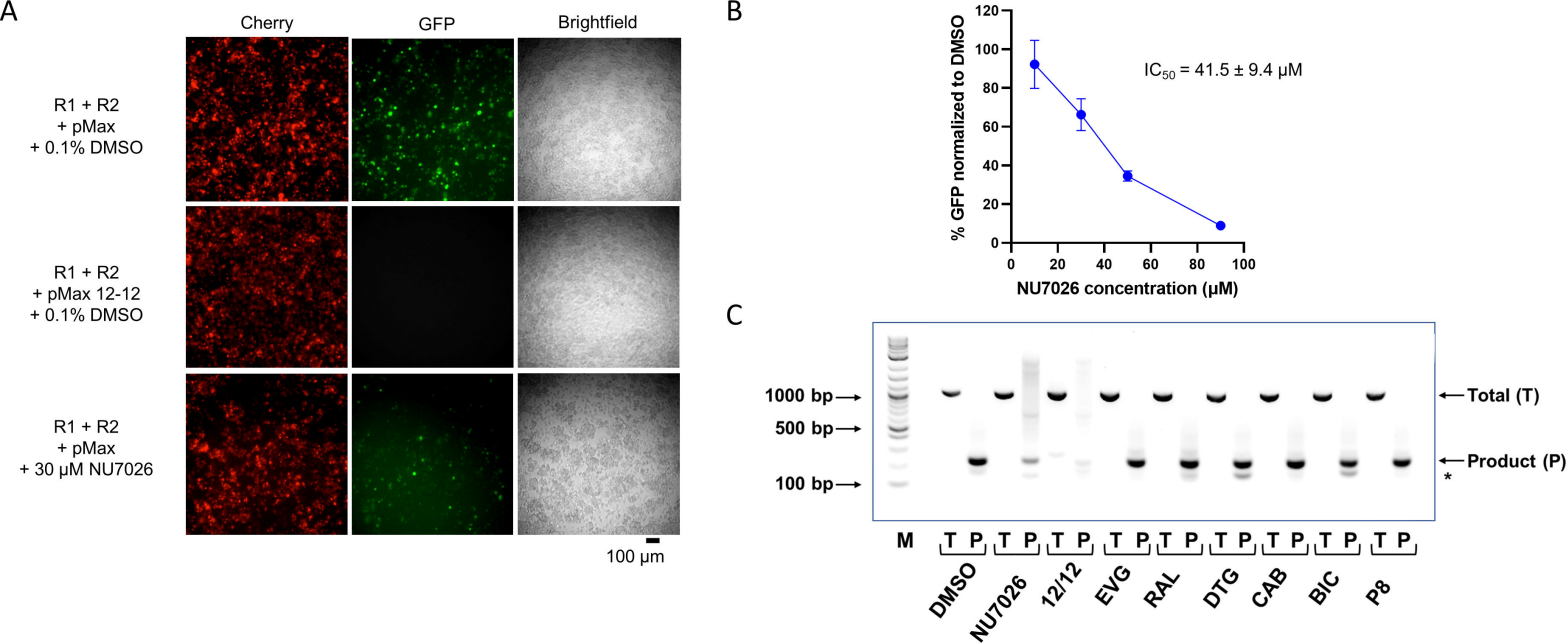
Validation of RAG1/RAG2 cellular assay. (**A**) Expi293F cells were co-transfected with three separate vectors, pMAXgfp-INV plasmid substrate, mCherry-core RAG1, and mCherry-RAG2 expression vectors. Inversional VDJ recombination on pMAX-INV substrate creates an in-frame GFP gene that is expressed as a reporter. The vehicle control (0.1% DMSO), added 2 h after transfection, served as the positive control for GFP expression. Two negative controls included (i) a modified pMAXgfp-INV substrate containing two 12RSSs, inefficiently recombined by the RAG proteins, and (ii) transfections with 30-µM NU7026 added 2 h after transfection. Transfection efficiency was evaluated using the mCherry fluorescence signal, and GFP expression was assessed 48 h post-transfection via microscopy. (**B**) Expi293F cells were co-transfected with pMAXgfp-INV plasmid substrate, mCherry-core RAG1, and mCherry RAG2, with 10-, 30-, 50-, and 90-µM NU7026 inhibitors added 2 h after transfection. Forty-eight hours post-transfection, the percentage of GFP-positive cells was determined by normalizing the flow cytometry data for each condition to the positive control (0.1% DMSO). Dose response in inhibition of GFP signal was observed for NU7026. (**C**) PCR of isolated plasmids with the recombined product shown in the sample containing both RAG proteins and treated with INSTIs (10 µM) but not in the negative control 12/12. DNA marker (M): TriDye 1 kb plus DNA marker; positions of 100-, 500-, and 1,000-bp markers are labeled; total (T): PCR product using primers that amplify both the unrearranged and rearranged plasmids; product (P): PCR product containing 12/23 signal Joint. *Non-specific PCR product.

For the recombination assay, the pMAXgfp-INV plasmid substrate and RAG1/RAG2 expression vectors (mCherry-core RAG1 and mCherry-RAG2) were transiently transfected into Expi293 cells in the absence or presence of integrase inhibitors and were assessed via microscopy and flow cytometry. The fluorescence signal from the mCherry-tag is used to assess transfection efficiency of RAG1/RAG2 vectors (Fig. 6A). We observed that transfection of the pMAXgfp-INV substrate into Expi293 cells with RAG1/RAG2 expression vectors yielded robust GFP expression 48 h post-transfection in the cells treated with vehicle control (0.1% dimethyl sulfoxide [DMSO]) (Fig. 6A). In contrast, GFP expression was not detectable in the negative control (pMAXgfp-INV plasmid substrate containing two 12RSSs), and lower GFP expression was observed in cells treated with the DNA-dependent protein kinase inhibitor, NU7026 (Fig. 6A). NU7026 inhibits double-strand break repair, which is a necessary step after DNA cleavage by RAG enzymes, and thus serves as an indirect inhibitor of RAG activity (35). Flow cytometry analysis to quantify the effect of NU7026 treatment on recombination activity showed a dose-dependent decrease in percentage of GFP-positive cells relative to the vehicle control (Fig. 6B). This demonstrates that RAG1/RAG2 can be inhibited in this assay by a non-specific inhibitor of V(D)J recombination and serves as a good control for testing integrase inhibitors in this system.

In addition, we isolated plasmid DNA from cells, selectively amplified, and sequenced the 12/23 signal joint to further confirm recombination. Agarose gel electrophoresis of the PCR products shows the signal joint PCR product at the correct size for the positive control and all integrase inhibitors tested at 10 µM (Fig. 6C). We observed a very faint PCR product for the negative control (pMAXgfp-INV plasmid substrate containing two 12RSSs), as expected. The positions of the PCR primers on the plasmids before and after recombination, as well as the sizes of the expected PCR products and primer sequences, are presented in Fig. S3; Table S1. The expected sequence of the precise 12/23 signal joint (from PCR product) was confirmed for all conditions, with representative results shown in Fig. S3. These results demonstrate that V(D)J recombination events resulting in 12/23 signal joint sequence is confirmed through DNA sequencing and occurred as expected even when cells were treated with the highest concentration of each integrase inhibitor.

### INSTIs do not inhibit RAG activity at clinical concentrations in a cellular assay

The microscopy and PCR data discussed above provided the necessary validation of this system to test the activity of RAG1/RAG2 in a cellular assay. Moreover, DNA sequencing confirmed the correct 12/23 signal joint sequence after V(D)J recombination even with the highest integrase inhibitor treatment. In order to perform a higher throughput and quantitative analysis of the effects of INSTIs on RAG recombination activity, we utilized flow cytometry. We optimized the assay conditions to determine the optimal timing for drug addition (before or after transfection with plasmids to avoid interfering with transfection efficiency) and total assay time or time after transfection (data not shown). The optimal conditions for GFP expression showing the highest signal-to-background ratio were the addition of vehicle or candidate inhibitors to cells 2 h after transfection and evaluation of GFP expression 48 h post-transfection using flow cytometry. Then, we tested six HIV-1 integrase inhibitors (Fig. 3) in at least three independent experiments at starting concentrations that are clinically relevant: 10 µM for all except p8. We took into consideration the observed human protein-unbound clinical maximum plasma concentrations (free *C*_max_) and potential cytotoxicity (Table 1). The CC_50_ values for these compounds in other cellular assays are listed in Table S2. The concentration of free compound used in these studies is supratherapeutic and expected to be much greater than the free concentration of licensed INSTIs at approved dosages in treated patients. In addition, the serum-free media used in our cellular assay resulted in much lower protein binding of the INSTIs than in the human serum, consequently leading to higher levels of free compound.

**Fig 7:**
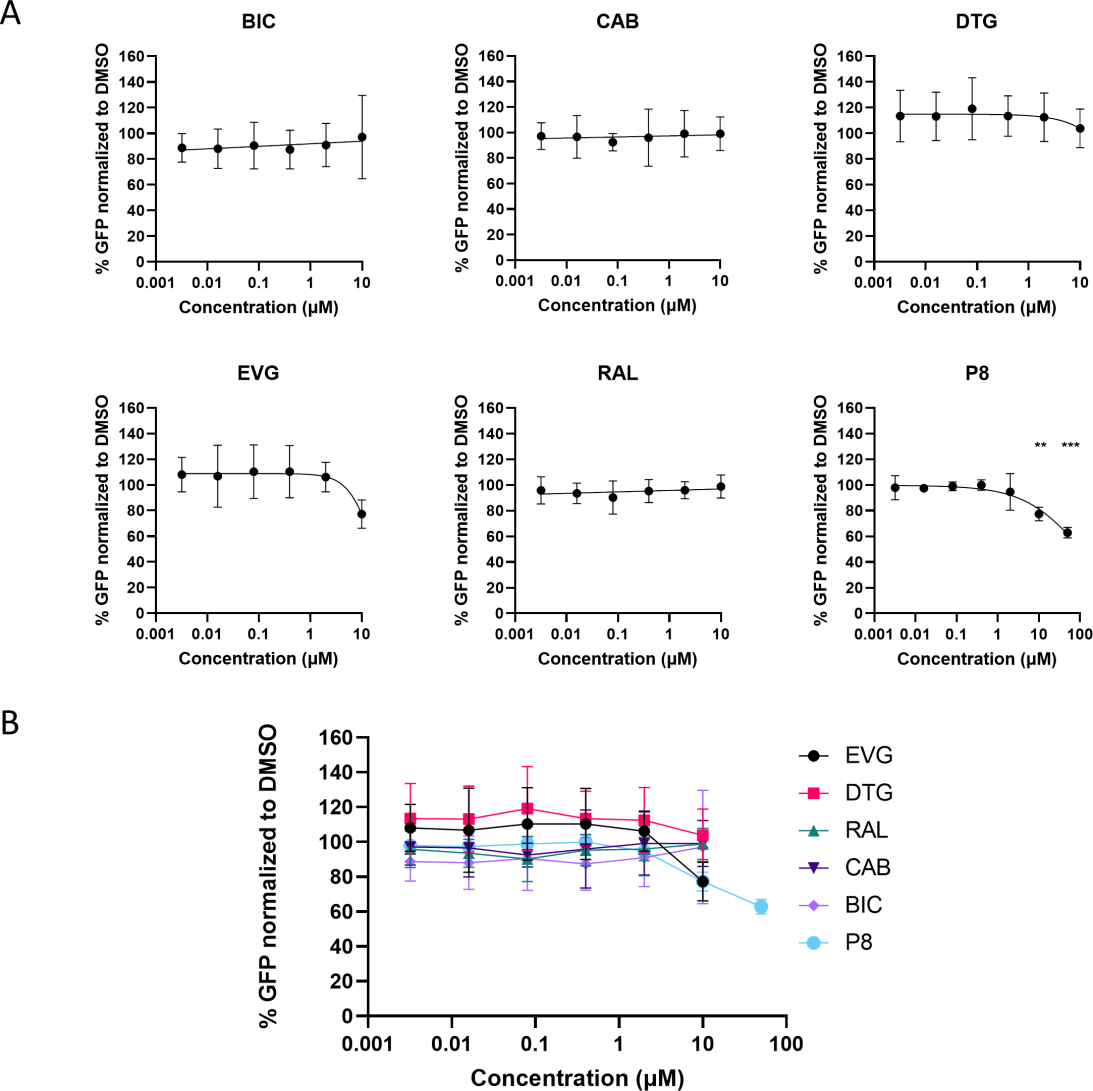
Assessment of cellular inhibition of RAG1/RAG2 recombination by INSTIs. (**A**) Expi293F cells were co-transfected with pMAXgfp-INV plasmid substrate, mCherry-core RAG1, and mCherry RAG2, with INSTIs added 2 h after transfection. Six different concentrations of each inhibitor, tested in fivefold dilutions starting from 10 µM (except for p8 starting from 50 µM), were added to separate wells. Forty-eight hours post-transfection, the percentage of GFP-positive cells for INSTIs tested was determined by normalizing the flow cytometry data for each condition to the positive control (0.1% DMSO). BIC (*n* = 5), CAB (*n* = 3), DTG (*n* = 3), EVG (*n* = 4), and RAL (*n* = 3) do not show inhibitory activity against RAG1/RAG2 at clinically relevant concentrations. p8 (*n* = 3) partially inhibited RAG1/RAG2 with an IC_50_ of >50 µM. An ordinary one-way analysis of variance with Dunnett’s multiple comparisons test was used to compare percentage of GFP levels to the DMSO control. **P ≤ 0.01, ***P ≤ 0.001. Unmarked datapoints denote non-significance. (**B**) Summary of data for all INSTIs tested in extrachromosomal V(D)J recombination assay.

The vehicle control (0.1% DMSO) served as the standard of 100% for GFP expression; therefore, the GFP-positive cells quantified by flow cytometry for each condition was normalized to the 0.1% DMSO conditions. Each experiment also included the controls, NU7026 (30 and 50 µM), and the pMAXgfp-INV substrate containing two 12RSSs. We expected the maximal percentage of GFP-positive cells in samples treated with vehicle only. If compounds inhibit V(D)J recombination, then we would expect a dose-dependent decrease in the percentage of GFP-positive cells that is significantly decreased relative to the vehicle-only treated cells. We observed a decrease of percentage of GFP-positive cells with increasing concentrations of p8. Although the maximum concentration of p8 we tested was 50 µM, constraining the data to assume 100% inhibition provides an IC_50_ estimation of 107 µM (Fig. 7), corroborating the findings within the literature and our biochemical data (7). For most of the HIV-1 integrase inhibitors tested in our cellular RAG assay (BIC, CAB, DTG, and RAL), we did not observe a reduction in percentage of GFP-positive cells (RAG inhibition) across the concentrations tested (Fig. 7). We observed a slight reduction in percentage of GFP-positive cells only at the highest concentration of EVG tested (~75% of the vehicle control). However, it should be noted that this concentration is ~455 times higher than the *C*_max_. To examine the cytotoxicity of integrase inhibitors in these cells, we used CellTiterGlo, a measurement of cellular ATP level, to quantify cell viability. The percentage inhibition of cell viability for all conditions was plotted in Fig. S4. Overall, based on these results, we do not expect that clinical concentrations of these integrase inhibitors will have off-target effects on recombination by RAG1/RAG2 in a cell-based V(D)J recombination assay. These data are also consistent with data generated from our biochemical assays.

## DISCUSSION

Advancements in HIV-1 treatments, particularly the use of INSTIs in combination antiretroviral therapy, have significantly improved the morbidity and mortality among PWH by suppressing the virus, preserving immune function, and preventing disease progression. If HIV-1 integrase inhibitors cause off-target inhibition of RAG recombination, this may subsequently lead to immunodeficiencies and immune dysregulation, including autoimmune disease in people with HIV-1. Here, we performed a comprehensive assessment of the off-target effects of INSTIs on RAG activity in biochemical and cellular assays at concentrations relevant to clinical exposure under approved dosages.

We investigated the effect of physiologically relevant concentrations of HIV-1 integrase inhibitors on RAG activity/V(D)J recombination activity using a biochemical assay and a novel extrachromosomal cell-based assay. At clinically relevant concentrations, biochemical assays showed no inhibition of DNA cleavage by INSTIs, and cellular assays similarly showed no significant RAG recombination inhibition by INSTIs. In comparison, p8, a prototype INSTI that did not advance into development due to toxicity, exhibited inhibition of RAG1/RAG2 in the biochemical assay similar to previous literature. Additionally, p8 showed signs of inhibition at concentrations greater than 10 µM in the cellular assay, which correlated with its observed toxicity. Therefore, we determined that the clinically approved INSTIs are unlikely to disrupt RAG-catalyzed DNA recombination through both biochemical and cellular methods.

To our knowledge, this is the first instance of a catalytically active human core RAG1–core RAG2 complex being purified and tested in the presence of human HMGB1, a co-factor known to enhance DNA cleavage *in vitro* and necessary for recombination *in vivo* (36). Historically, the field has focused on using murine RAG1/RAG2 for *in vitro* studies based on their availability (37, 38). In this study, we showed that biochemical assays using recombinant human enzymes are in agreement with the cellular assays using engineered murine core RAG1/FL RAG2, consistent with the high sequence homology (~90%) between human and mouse RAG including identical catalytic residues. Using two orthogonal approaches, we demonstrated that INSTIs are unlikely to have off-target effects on RAG.

RAG deficiencies can lead to severe combined immunodeficiency (SCID), Omenn syndrome, atypical SCID, or combined immunodeficiency with granulomas and/or autoimmunity (10, 39, 40). Thus, it is possible that inhibition of RAG1 by small molecules could result in similar significant immunodeficiency or the development of autoreactive T cells, leading to autoimmune disorders. However, such outcomes have not been observed in the pre-clinical and clinical settings for INSTIs to date, and there is no evidence in published literature that inhibition of RAG1 activity by any INSTI would lead to a decline in CD4 cell numbers. As an example, mice treated with EVG did not show a large decrease in T or B cells (9). Moreover, juvenile rat studies using DTG showed no impact on immunological competence when assessing T-cell receptor (TCR) Vβ usage by flow cytometry as an indicator of TCR repertoire diversity and T cell-dependent antibody response, an indicator of immunosuppression (41). Furthermore, an *in utero* study was performed by assessing the PROMIDISα signature, a next-generation sequencing method that measures impaired V(D)J recombination, on newborns born to women with HIV-1 who were taking antiviral therapies consisting of INSTIs (42). In this study, the diversity of T-cell receptor α did not differ from newborns exposed to INSTIs compared to the control group (non-INSTI antivirals). These data support that INSTIs would not be expected to impact RAG1 activity *in vivo*.

*In vitro* findings through biolayer interferometry by Nishana et al. reported a dissociation constant for EVG from RAG1 at 32.5 µM (9). Comparatively, data assessing the equilibrium dissociation constants for EVG and other approved INSTIs from an integrase–viral DNA complex ranged in the low nanomolar range (~3 to 9 nM) (30–32) (Table 1), showcasing a ≥1,000-fold difference. Hence, EVG and other INSTIs exhibit significantly higher affinity for binding to HIV-1 integrase–viral DNA complex compared to human RAG1, establishing high selectivity and contributing to overall drug class safety.

When considering the potential interaction between INSTIs and RAG1, it is crucial to recognize the highly specific binding of INSTIs with HIV-1 integrase from a structural viewpoint. INSTIs bind to a pair of catalytic magnesium ions which are coordinated by a DDE motif (Asp64, Asp116, and Glu152) within the integrase active site. This interaction is critical but not sufficient in isolation to achieve high-affinity binding and inhibition. Recent high-resolution structural studies of BIC (3) and DTG (43) binding to the HIV-1 intasome emphasize the importance of additional specific interactions between these INSTIs and the HIV-1 integrase catalytic site and viral DNA. While the catalytic site of RAG1 also possesses a DDE motif (Asp603, Asp711, and Glu965), which will coordinate two catalytic metal ions (15), RAG1 and HIV-1 integrase are otherwise dissimilar with no sequence conservation and no meaningful structural alignment, in contrast to the conclusions proposed by Nilavar and Raghavan (44). Indeed, an examination of multiple available structures of the highly homologous mouse RAG1–RAG2 complex in various states (45) clearly demonstrates fundamental differences in the nature of the catalytic site and how it interacts with DNA, leading to the conclusion that INSTIs simply cannot bind to RAG1 with the same specificity that they do to HIV-1 integrase (46, 47).

In conclusion, utilizing both biochemical and cellular assays, this study reveals that at clinically relevant concentrations, the tested INSTIs exhibit no relevant off-target effects on RAG recombinase activity. The findings support the continued use of the highly selective INSTIs in HIV-1 treatment without substantial concern for adverse effects on V(D)J recombination and immune system integrity.

## MATERIALS AND METHODS

### Reagents and chemicals

Integrase inhibitors were synthetized at Gilead Sciences, Inc. (Foster City, CA, USA), including BIC, CAB, DTG, EVG, and RAL (> 95% purity). The prototype integrase inhibitor p8/5-CITEP was synthesized by Enamine (Kiev, Ukraine) (95% purity). Suramin, a non-specific inhibitor in the biochemical RAG assay, was purchased at Alfa Aesar (Haverhill, MA, USA). The DNA-dependent protein kinase inhibitor, NU7026 (ab120970), was purchased at Abcam (Cambridge, UK) (>99% purity) and served as a negative control for GFP expression in the extrachromosomal cell-based V(D)J recombination assay.

### Plasmids

Human RAG1 core-domain gene sequence encoding amino acid V387-A1011 was cloned into pcDNA3.1 in frame with N-terminal MBP, 10 asparagine residues, and PreScission protease (MBP-10N-PreScission-RAG1) as described previously (45, 48). Similarly, the human RAG2 core-domain gene sequence encoding amino acid M1-S387 was cloned into pcDNA3.1 with N-terminal MBP and five alanine residues (MBP-5A-RAG2). A truncated form of the human HMGB1 gene encoding amino acid M1-R163 was cloned into pET21a vector in frame with N-terminal 6xHis, and tobacco etch virus (TEV) protease His-TEV-HMGB1 (M1-R163) (49).

### Expression and purification of human proteins (RAG1/RAG2 and HMGB1)

#### Human core RAG1 (AA387-1011)–core RAG2 (AA1-387) complex

Expression of the RAG1–RAG2 complex was achieved by transfecting plasmids into human cells, Expi293F GnTI, using polyethylenimine hydrochloride (PEI MAX) transfection reagent (Polysciences Inc., Warrington, PA, USA). Cells were harvested 96 h post-transfection and stored at –80°C. For protein purification, the cell pellet was resuspended in Buffer A (25-mM Tris-HCl [pH 7.5], 500-mM NaCl, 2-mM dithiothreitol [DTT] supplemented with 1-mM MgCl_2_, benzonase nuclease [3 U/mL], and Roche cOmplete Ethylenediaminetetraacetic Acid [EDTA]-free Protease Inhibitor Cocktails [Sigma-Aldrich, St. Louis, MO, USA]). The cell suspension was passed through a microfluidics high-pressure homogenizer. The cell lysates were centrifuged at 16,000 rpm for 30 min at 4°C, and the supernatant was loaded over an MBP-Trap high-performance (HP) column (Cytiva, Marlborough, MA, USA). The column was washed with Buffer A, and the protein was eluted in the same buffer containing 10-mM maltose. Subsequently, the purified sample was loaded over a Superdex 200 Increase 10/300 column equilibrated with 50-mM HEPES (pH 7.0), 500-mM KCl, 5% glycerol, 2-mM DTT, and 2-mM maltose. The RAG1–RAG2 complex was eluted in two peaks. The lower MW second peak that was eluted later (Fig. S1D) was collected and adjusted to a final glycerol concentration of 30% in the same buffer. All preparative protein purification steps described above were carried out using an AKTA PURE fast performance liquid chromatography (FPLC) system (Cytiva). Analytical scale (10-µg protein sample) experiments were carried out using the 1260 Infinity II high-performance liquid chromatography (HPLC) system (Agilent Technologies, Santa Clara, CA, USA) using a 40-bar column pressure limit.

#### Human HMGB1 (AA1-163)

Expression of the recombinant human HMGB1 (AA1-163) protein was achieved by transforming the plasmid into *E. coli* BL21DE3 (Agilent) cells and inducing *E. coli* culture in lysogeny broth (LB) media with isopropyl β- d-1-thiogalactopyranoside (IPTG) (Sigma-Aldrich) to a final concentration of 1 mM at 30°C for 4 h. Induced culture was harvested and stored at –80°C. For HMGB1 protein purification, the cell pellet was resuspended in lysis buffer (40-mM Tris–HCl [pH 7.5], 500-mM NaCl, 0.1-mM EDTA, 0.25% Tween 20, 5% glycerol, and 20-mM imidazole) supplemented with Roche cOmplete EDTA-free Protease Inhibitor Cocktails (Sigma-Aldrich). The cell suspension was passed through a microfluidics high-pressure homogenizer. The cell lysates were centrifuged at 16,000 rpm for 30 min at 4°C, and the supernatant was loaded over a HisTrap excel column (Cytiva) equilibrated with Buffer A (40-mM Tris–HCl [pH 7.5], 500-mM NaCl, 0.1-mM EDTA, 5% glycerol, and 1-mM phenylmethylsulfonyl fluoride [PMSF]). The target protein was eluted using Buffer B (40-mM Tris–HCl [pH 7.5], 500-mM NaCl, 0.1-mM EDTA, 5% glycerol, 1-mM phenylmethylsulfonyl fluoride [PMSF], and 0.5-M imidazole). The eluted sample was cleaved with TEV protease overnight at 4°C, desalted to Buffer A, and purified by reverse affinity chromatography using the HisTrap Excel column. Both flow-through and 20-mM imidazole wash samples were collected, buffer exchanged, and loaded over a HiTrap Heparin HP column (Cytiva) equilibrated with Buffer C (25-mM Tris–HCl [pH 7.5], 0.1-mM EDTA, 1-mM DTT, 5% glycerol, and 100-mM NaCl). The target protein was eluted by a linear gradient using Buffer D (25-mM Tris–HCl [pH 7.5], 0.1-mM EDTA, 1-mM DTT, 5% glycerol, and 600-mM NaCl). Most pure fractions were further polished using a Superdex 75 Increase 10/300 Gl column (Cytiva) equilibrated in storage buffer (25-mM HEPES [pH 7.0], 100-mM KCl, 2-mM DTT, 0.1-mM EDTA, and 5% glycerol). All preparative purification steps and subsequent small-scale analytical experiments were carried out using FPLC and HPLC systems, respectively, as described for RAG1/RAG2 proteins.

#### Multi-angle light scattering

The purified HMGB1 (AA1-163) protein sample (1.37 µg/µL in 55-µL volume) was applied over a Superdex 200 Increase 5/150 Gl column (Cytiva) equilibrated in 25-mM HEPES (pH 7.0), 100-mM KCl, 2-mM DTT, and 0.1-mM EDTA at a flow rate of 0.3 mL/min. The column was coupled with multi-angle light scattering equipment, and scattered light intensity (refractive index) of the column eluate (elution volume) was recorded using a DAWN-Optilab instrument (Wyatt Technology, Santa Barbara, CA, USA). The molecular mass of the eluent was computed using ASTRA software (version 8.1.2, Wyatt Technology). All the measurements were performed at room temperature.

#### Intact LC–MS

An Agilent 6230 time-of-flight LC/MS system was employed to measure protein intact mass using a MAbPac Phenyl reverse-phase column (Thermo Fisher Scientific, Waltham, MA, USA). For LC, a gradient with water/acetonitrile/formic acid was used as the mobile phase. About 500- to 1,000-ng protein samples were used for each analysis.

### dsDNA substrate design and production

A heterogeneous dsDNA substrate containing both the 12RSS and 23RSS was designed for biochemical RAG1/RAG2 cleavage assays. A 569-bp double-stranded fragment was inserted into a pUC57 vector (GenScript, Piscataway, NJ, USA) (Table S3). When fully cleaved by RAG1/RAG2, the 569-bp dsDNA substrate yielded 77-, 136-, and 356-bp-length products under native gel conditions.

To synthesize the dsDNA substrate from the pUC57 plasmid, forward and reverse PCR primers were designed to amplify the 569-bp segment. OneTaq 2× MasterMix (New England Biolabs, Ipswich, MA, USA) was used to amplify the substrate (Table S4). A typical 50-µL reaction contained 500-nM forward and reverse primers, 10 ng of the pUC57 plasmid, and 1× of the MasterMix. The PCR thermocycling conditions were as follows: (i) initial denaturation at 94°C for 30 s for one cycle; (ii) 94°C for 15 s, 56°C for 30 s, and 68°C for 45 s for 30 cycles; and (iii) 68°C for 50 min for one cycle. The completed reactions were resolved by 1% agarose gels at 100 V for 1 h. The 569-bp dsDNA product band was detected via a UV transilluminator and purified via the QIAquick gel extraction kit (Qiagen, Redwood City, CA, USA). To further purify the substrate, the DNA was ethanol precipitated and redissolved in distilled H_2_O. A 374-bp standard used as a marker for RAG cleavage assays was synthesized from the same vector and purified with this method using alternative PCR primers to produce a 374-bp dsDNA.

### DNA cleavage by recombinant human RAG1/RAG2–HMGB1 complex and data analysis (biochemical assay)

Prior to the cleavage assay, compounds at the designated concentrations were spotted into 96-well plates using the Tecan Digital D300e (Tecan, Mannedorf, Switzerland). RAG1/RAG2 was incubated with HMGB1 and MgCl_2_ on ice for at least 15 min in a reaction buffer (25-mM 3-(N-morpholino)propanesulfonic acid [MOPS], pH 7.0, 50-mM KCl, and 2-mM DTT). After the initial incubation, the enzyme solution was added to the compound plates and incubated for an additional 15 min at room temperature. dsDNA substrate in reaction buffer was added to the plates to initiate the reaction. The final conditions of the reaction are 25-nM RAG1–RAG2 complex, 575-nM HMGB1, 10-nM dsDNA substrate, and 5-mM MgCl_2_. After 1 h at 37°C, reactions were quenched by the addition of final concentrations of 0.19% SDS, 45.5-µg/mL Proteinase K, and 60-mM EDTA.

Glycerol was added to a final concentration of 7.6% in each sample, and samples were loaded onto non-denaturing 6% Tris–borate–EDTA (TBE) gels in 1× TBE buffer. The samples were run at 100 V for 1.5 h, and gels were stained with 1× SYBR Gold in 1× TBE for 20 min. The stained gels were imaged with an Amersham Typhoon Biomolecular Imager.

The gel images were analyzed using ImageQuant software (Cytiva). The densities of the 569-bp-length substrate and fully cleaved 356-bp-length product were quantified. The product density was divided by the sum of substrate and product bands to obtain a percent product value. These calculated values were subtracted from a negative control (pre-quenched reaction where EDTA was added before reaction was initiated) and then normalized to the DMSO-treated control. The normalized data were plotted against concentration using GraphPad Prism (version 9.3; GraphPad Software, San Diego, CA, USA).

### RAG expression protein vectors for cellular assay

The RAG expression vectors encode for the catalytically essential core region of murine RAG1 (residues 384–1008 of the 1,040-residue full-length murine protein) and full-length murine RAG2 expressed as C-terminal fusions to mCherry, termed mCherry-core RAG1 and mCherry-RAG2, respectively. Construction of the vector encoding for mCherry-RAG2 was previously described (50). The mCherry-core RAG1 vector was constructed by inserting the murine RAG1 gene segment encoding for the core region into the pmCherry-C1 vector (Takara Bio USA, San Jose, CA, USA) using NEBuilder Assembly (New England Biolabs). The parent construct pmCherry-C1 and the core RAG1 gene region were amplified with “ChVec-f + ChVec-r” and “cR1-f + cR1r” primers (Integrated DNA Technologies [IDT], Coralville, IA, USA), respectively (Table S5). The corresponding PCR products were assembled with NEBuilder HiFi DNA Assembly kit (NEB) following manufacturer instructions. The sequence of the assembled vector containing the gene encoding for mCherry-core RAG was confirmed by whole-plasmid sequencing (Plasmidsaurus, Eugene, OR, USA).

### pMAXgfp-INV extrachromosomal recombination substrate for cellular assay

A plasmid substrate (pMAXgfp-INV) was designed with a 12RSS and a 23RSS flanking each side of the *Pontellina plumata* green fluorescent protein gene (termed maxGFP), where the gene is in an inverted orientation relative to the CMV promoter (described in reference [33]). The 12RSS and 23RSS sequences are 5′-CACAGTGCTACAGACTGGAACAAAAACC-3′ and 5′-CACAGTGGTAGTACTCCACTGTCTGGCTGTACAAAAACC-3′, respectively. The RSSs are positioned in a co-linear orientation, such that successful V(D)J recombination activity will result in inversion of the maxGFP gene to place it in a transcriptionally active orientation with resulting expression of GFP. Detection of GFP-expressing cells was used as a direct readout of V(D)J recombination activity.

### Cell culture and maintenance

Expi293F cells (Gibco, Carlsbad, CA, USA) are suspension-adapted human embryonic kidney 293 cells cultured in Gibco Expi293 expression medium. Expi293 expression medium is a serum-free, protein-free, chemically defined medium formulated with GlutaMAX-I reagent for optimal growth and transfection of these cells. Expi293F cells were maintained in suspension culture on a Thermo Fisher Scientific CO_2_ resistant shaker in a standard 8% CO_2_ humidified incubator at 37°C. Cells were maintained at subconfluency and passaged every 2–3 days.

### Extrachromosomal cell-based V(D)J recombination assay and data analysis

To test the effect of HIV-1 integrase inhibitors on V(D)J recombination activity, we optimized an extrachromosomal cell-based V(D)J recombination assay (33). Briefly, Expi293 cells were plated at 1 × 10^6^ cells/mL in six-well plates in Gibco Expi293 expression media the day before transfection. The next day, the Expi293 cells were co-transfected with three separate vectors, pMAXgfp-INV plasmid substrate, mCherry-core RAG1, and mCherry-RAG2 expression vectors with a 4:1 transfection reagent:DNA ratio using ExpiFectamine 293 transfection reagent (Gibco). Vehicle or candidate inhibitors were added to cell samples 2 h after transfection, with six different concentrations of each inhibitor added to separate wells. Fivefold dilutions for each inhibitor starting from 10 µM were tested.

Experiments were performed with six separate inhibitors in at least three independent experiments. Two negative control conditions for recombination activity were performed in parallel and include (i) transfections in the presence of NU7026 at 30 and 50 µM (Abcam) and (ii) using a modified pMAXgfp-INV substrate containing two 12RSSs (instead of one 12RSS and one 23RSS) that is inefficiently recombined by the RAG proteins. Vehicle control (0.1% DMSO) served as the positive control for GFP expression. The fluorescence signal from the mCherry tag was used to assess transfection efficiency. GFP expression was assessed 48 h post-transfection using the Cytek Aurora for flow cytometry (Cytek Biosciences, Fremont, CA, USA) at the Oklahoma Medical Research Foundation (OMRF) flow cytometry facility. Cell viability was quantified for each cell sample by flow cytometry using Zombie staining (BioLegend, San Diego, CA, USA) and CellTiterGlo (Promega, Madison, WI, USA) (described below). Live cells were gated based on a negative signal from Zombie staining, followed by gating on mCherry-positive cells. GFP-positive cells were quantified from the live, mCherry-positive cell population. The percentage of GFP-positive cells was determined by normalizing the flow cytometry data for each condition to the positive control (0.1% DMSO).

The normalized data were plotted against concentration and fit to an inhibition dose–response equation using GraphPad Prism (version 9.3, GraphPad Software). Estimation of the p8 IC_50_ was determined by constraining the bottom of the equation to 0%. This constraint assumes that full inhibition can be achieved at compound concentrations higher than those tested.

### Cell viability assay

After 48-h incubation at 37°C in a humidified 8% CO_2_ incubator, 100 µL of cell suspension was transferred to 96-well black assay plates (Corning). Each well of the assay plates then received 100 µL CellTiterGlo reagent and the resulting luminescence signal was read using the PHERAstar FSX plate reader (BMG Labtech, Germany). The luminescence data were normalized to the vehicle control, 0.1% DMSO (set to 100%), and the percentage inhibition of cell viability for all conditions was plotted using Prism (version 9; GraphPad Software, La Jolla, CA).

### Testing signal joint formation by PCR and DNA sequencing in cells

Plasmid DNA was isolated from cells in selected conditions, and signal joints present in the recovered plasmid were selectively amplified by PCR. The PCR amplicon was purified by agarose gel electrophoresis and subsequently sequenced by Sanger sequencing.

Briefly, after the 48-h incubation time point in the extracellular recombination assay, 1 mL of cells from the vehicle control (0.1% DMSO), from negative controls (NU7026 and pMAXgfp-INV substrate containing two 12RSSs), and from cell samples treated with 10-µM inhibitor was frozen at –20°C. Plasmid DNA was subsequently isolated from each sample according to a previously published protocol (51), and the concentration of the purified plasmid DNA was quantified using a Qubit fluorometer (Thermo Fisher Scientific). To detect inversional recombination events containing a 12/23 signal joint, end-point PCR was performed. PCR reactions (at 50 µL) contained 10 ng of plasmid DNA, 0.5 µM of Max-forward and Max-reverse primers (IDT, Table S1), 200-µM dNTPs, and 0.02 units/µL of Q5 Hot Start High-Fidelity DNA polymerase (NEB). The PCR thermocyler conditions are as follows: (i) initial denaturation and activation of the HotStart polymerase at 98°C for 30 s for one cycle; (ii) 98°C for 10 s, 63°C for 30 s, and 72°C for 1 min for 25 cycles; and (iii) 72°C for 2 min for one cycle. PCR products were resolved by electrophoresis on 1% agarose gels in 1× Tris acetate EDTA running buffer (40-mM Tris, 20-mM acetic acid, 1-mM EDTA, pH 8.3), and DNA bands stained with GelGreen DNA stain were visualized with a ChemiDoc imaging system (Bio-Rad, Hercules, CA, USA). Gel bands corresponding to PCR products containing the 12/23 signal joint were visualized by a blue light transilluminator and excised from the 1% agarose gel. DNA was extracted from the gel using the Monarch DNA Gel Extraction kit (NEB) according to the manufacturer’s protocol. The resulting DNA concentration was quantified using a Qubit fluorometer, and the DNA sequence was determined by Sanger DNA sequencing (using the Max-forward oligonucleotide as sequencing primer) performed at the OMRF DNA sequencing facility. PCR and DNA sequencing of the 12/23 signal joint confirmed recombination in the presence of both RAG proteins.

### Statistics (cell-based assay)

Statistical tests were performed in GraphPad Prism (version 9) and are described in figure legends where applicable. An ordinary one-way analysis of variance with Dunnett’s multiple comparisons test was performed when more than two groups were compared. Asterisks denote *P* value range, where *P ≤ 0.05, **P ≤ 0.01, ***P ≤ 0.001, and unmarked data denote non-significance.

## SUPPLEMENTAL MATERIAL

Supplemental material spectrum.02468-24-s0001.pdf Tables S1 to S5; Fig. S1 to S4.

ASM does not own the copyrights to Supplemental Material that may be linked to, or accessed through, an article. The authors have granted ASM a non-exclusive, world-wide license to publish the Supplemental Material files. Please contact the corresponding author directly for reuse.
