# Supplementary material for: Lack of activity of HIV-1 integrase strand-transfer inhibitors on recombinase activating gene (RAG) activity at clinically relevant concentrations
Source: Microbiol Spectr. 2024 Nov 19;13(1):e02468-24. doi: 10.1128/spectrum.02468-24 (PMC11705955; doi:10.1128/spectrum.02468-24)
Supplement: Supplemental material — Tables S1 to S5; Fig. S1 to S4. [file spectrum.02468-24-s0001.pdf]

SUPPLEMENTAL MATERIALS

Supplemental Table S1. Primer sequences to test signal joint formation in extracellular V(D)J recombination assay

Primer name	Primer sequence
T (Total) Max FWD	CCACTTTGCCTTTCTCTCCACAGG
T (Total) Max INPUT	TAACGTCTCGCCCTTTGGTCTCC
P (Product) Max FWD	CCACTTTGCCTTTCTCTCCACAGG
P (Product) Max RVS	TGCTCTTCATCTTGTTGGTCATGCGGC

T (Total) Max: PCR primers that amplify both the unrearranged and rearranged plasmids. P (product) Max: PCR primers that amplify the 12/23 Signal Joint.

Supplemental Table S2. CC_{50} values for INSTIs in 5-day cytotoxicity assays with Cell Titer Glo readout

Drug	CC_{50} (µM)							
	**Expi293F**	**GALPC3**	**GALHEP G2**	**HUH7**	**MRC5**	**MT4**	**PBMC Lot1**	**PBMC Lot2**
	Kidney (embryonic) cell line	Prostate cancer cell line	Hepatoma cell line	Hepatoma cell line	Lung fibroblast cell line	T-cell line	Primary cells	Primary cells
BIC	46.04	>44.44	17.39	38.01	>44.44	2.59	28.49	24.20
CAB	>100	>44.44	37.91	21.46	28.07	5.25	30.01	29.53
DTG	53.65	>44.44	>44.44	28.72	38.16	7.53	28.43	17.90
EVG	32.50	20.82	9.02	20.36	14.26	3.75	14.62	14.06

RAL	>100	>44.44	>44.44	>44.44	>44.44	>50	>44.29	>44.29
p8	>100	>44.44	>44.44	>44.44	>44.44	>50	>44.29	>44.29
Puromycin (control)	N/A	0.39	0.41	0.32	0.31	0.21	0.54	0.50

Supplemental Table S3. Sequence of pUC57 dsDNA substrate plasmid

**Sequence of pUC57 dsDNA substrate plasmid**
…CAGTGAATTCCACGATGCCATTGGGATATATCAACGGTGGTATATCCAGTGATTTT<br>TTTCTCCATTTTAGCTTCCTTAGCTCCTGTA**CACAGTGCTACAGACTGGAACAAAA**<br>**ACC**CTGCAGACAGCCAGACAGTGGAGTACTACCACTGTGGGATCCTCTCATCGATG<br>AGAGGATCGACGACGACATGGCTCGATTGGCGCGACAAGTTGCTGCGATTCTCACC<br>AATAAAAAACGCCCGGCGGCAACCGAGCGTTCTGAACAAATCCAGATGGAGTTCT<br>GAGGTCATTACTGGAGACCTGCCGAGTGGTTCAGCAGGTGGAAGAGGGACTGGAT<br>TCCAAAGTTCTCAATGCTGCTTGCTGTTCTTGAATGGGGGGTCGTTGATCCCCCATC<br>GATGAGAGTCGTAGGTTTTTGT**ACAGCCAGACAGTGGAGTACTAC**CACTGTGTAG<br>CAGTCGACCTGCAGCCCAAGCTTGGCGTAATCATGGTCATAGCTGTTTCCTGTGTG<br>AAATTGTTATCCGCTCACAATTCCACACAACATACGAGCCGGAAGCATAAAGTGTA<br>AAGCCTGGGGTGCCTAATGAGTGAGCTAACT…

Partial sequence of pUC57 vector containing the RAG1/RAG2 biochemical substrate. The bolded regions are the 12- and 23RSS. The underlined regions are the positions where the PCR primers bind to synthesize the linear dsDNA substrate.

Supplemental Table S4. Primer sequences for RAG1/RAG2 Biochemical Assay

**Primer name**	**Primer sequence**
Substrate-f	CACGATGCCATTGGGATATATCAACGG
Substrate-r	CTCATTAGGCACCCCAGGCTTTAC
374marker-f	CAACGGTGGTATATCCAG
374marker-r	CTACGACTCTCATCGATG

PCR was performed using a pUC57 vector to synthesize and amplify a linear dsDNA substrate and a 374 bp reference marker.

Supplemental Table S5. Primer sequences for mCherry-core RAG1 vector construction

**Primer name**	**Primer sequence**
ChVec-f	GAATGCTCATAACGCGTAACAAGCTTCGAATTCTGCAG
ChVec-r	CCTTTATTGATATGCACTCCGCCTCCGGACTTGTACAGCTC
cR1-f	TGTACAAGTCCGGAGGCGGAGTGCATATCAATAAAGGGG
cR1-r	ACTGCAGAATTCGAAGCTTGTTACGCGTTATGAGCATTC

The primer sequences for the construction of the mCherry-core RAG1 vector are listed in Supplemental Table 5.

SUPPLEMENTAL FIGURES

Supplemental Figure S1. Quality control analysis of human RAG1/RAG2 and HMGB1 recombinant proteins.

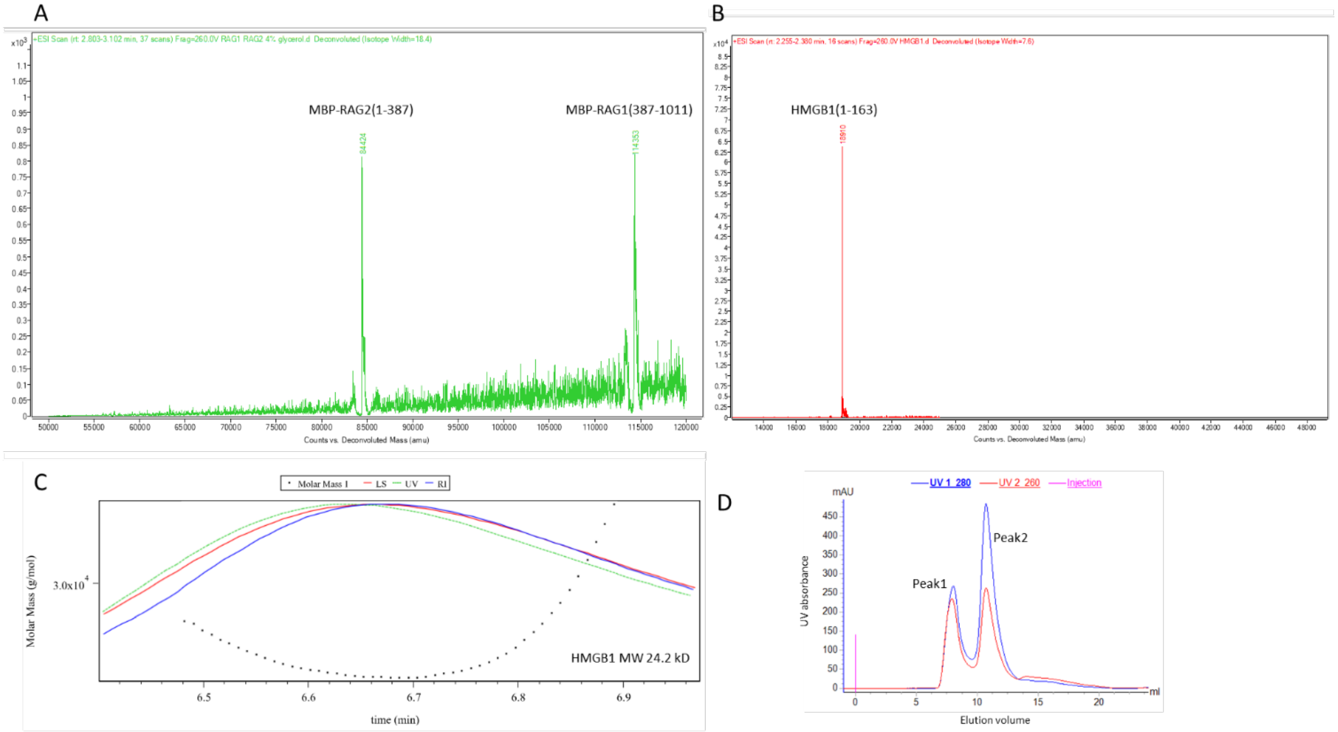

(A) Intact LC-MS analysis to verify the MW of the MBP-tagged human RAG1 and RAG2 proteins. (B) Intact LC-MS analysis to verify the MW of un-tagged human HMGB1 protein. (C) SEC-MALS analysis to determine the molar mass of HMGB1 (AA1-163) protein. (D) Superdex 200 Increase 10/300 column size exclusion chromatography purification of RAG1/RAG2 protein complex.

Supplemental Figure S2. Optimization of enzyme and buffer components of human RAG1/RAG2 biochemical assay.

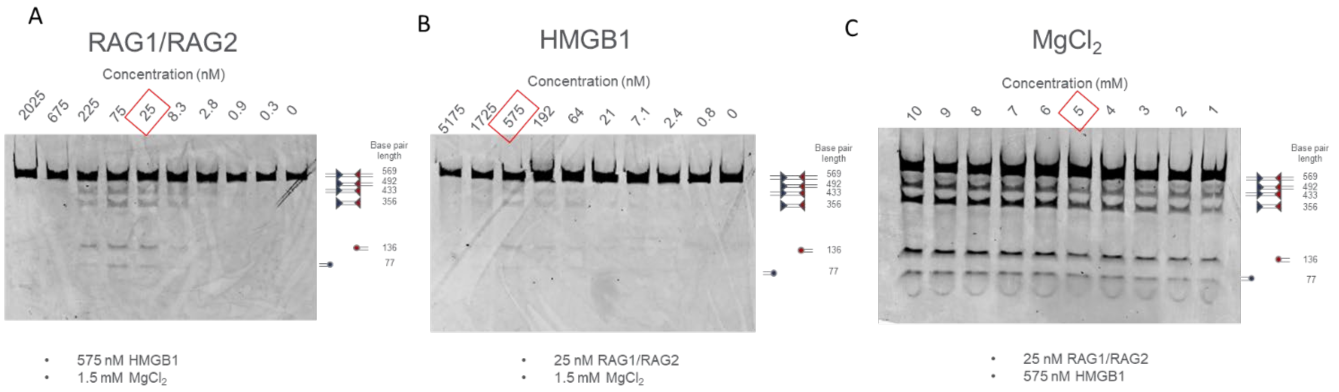

(A) RAG1/RAG2 were titrated with a 3-fold dilution scheme starting from 2.025 µM. (B) HMGB1 was titrated with a 3-fold dilution scheme with a starting concentration of 5.175 µM. (C) MgCl2 was titrated in increments of 1 mM with concentrations ranging from 1-10 mM. The final assay components were 25 nM RAG1/RAG2, 575 nM HMGB1, 5 mM MgCl2, and 10 nM DNA substrate.

Supplemental Figure S3. PCR and sequencing of signal joint formation.

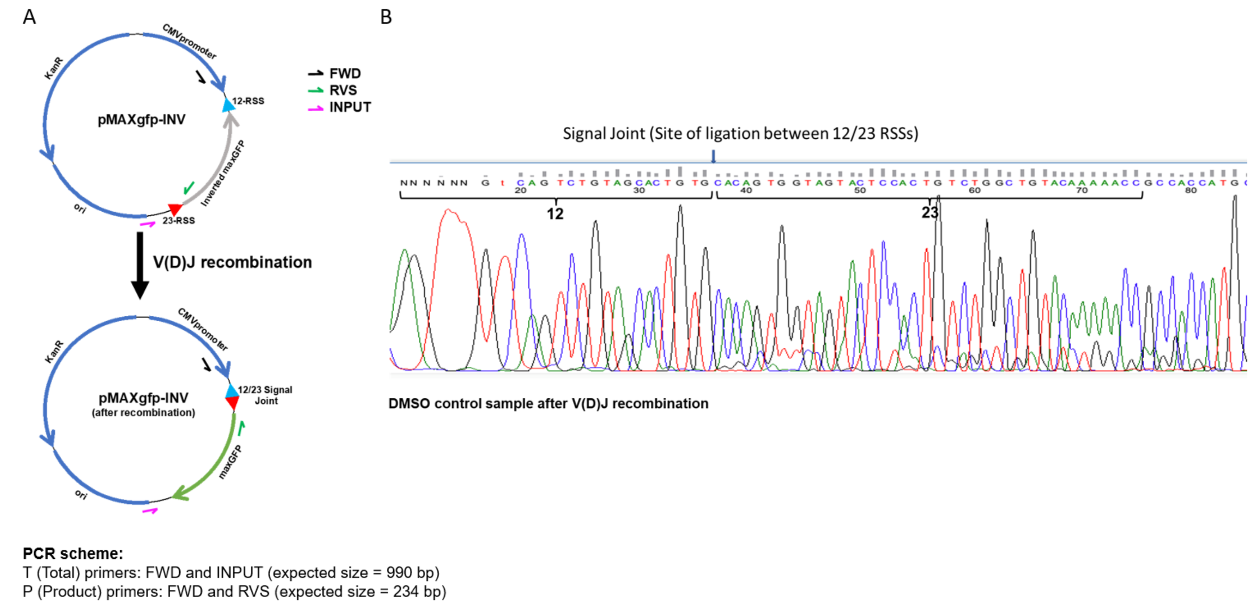

(A) Scheme of primer positions for PCR amplification of signal joint sequence before and after V(D)J recombination. FWD, RVS, INPUT primers are indicated on the figure. T (Total) primers: FWD and INPUT (expected size = 990 bp) P (Product) primers: FWD and RVS (expected size = 234 bp) (B) Sanger DNA sequencing of the DMSO control V(D)J recombination product (labeled as P in Figure 6 in the main text). As expected, V(D)J recombination resulted in a precise heptamer-to-heptamer signal joint between the 12RSS and 23RSS, as labeled in the figure above. PCR band 'P' in Figure 6 was gel purified using the Monarch DNA Gel Extraction Kit (#T1020S; New England Biolabs), and sequenced by Sanger sequencing at the Oklahoma Medical Research Foundation DNA sequencing facility. Only a portion of the 12RSS is resolved as the 12RSS is near the sequencing primer site.

Supplemental Figure S4. Cytotoxicity analysis of INSTIs in the cellular RAG assay using Cell-Titer Glo.

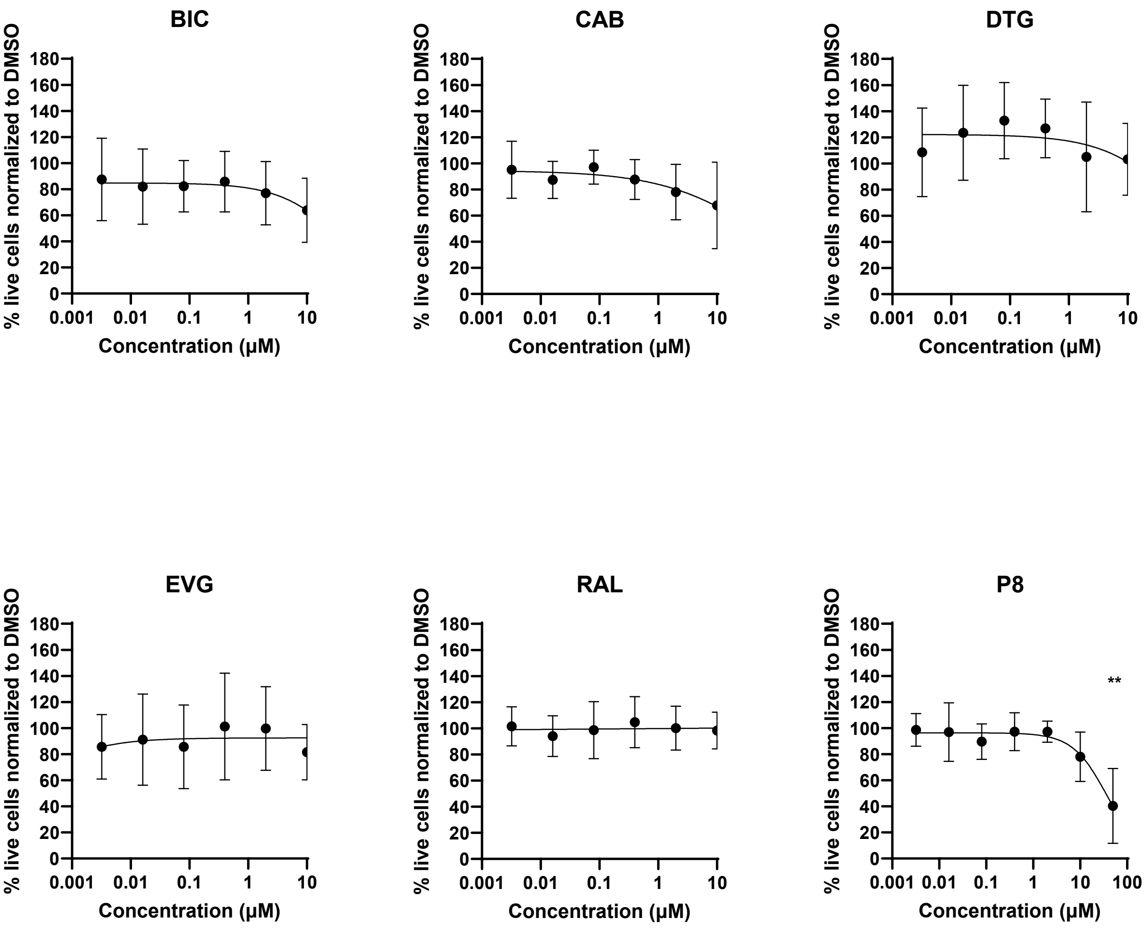

Expi293F cells were co-transfected with pMAXgfp-INV plasmid substrate, mCherry-core RAG1, mCherry RAG2, with INSTIs added 2 hours after transfection. Six different concentrations of each inhibitor, tested in 5-fold dilutions starting from 10 μM (except for p8 starting from 50 μM), were added to separate wells. % live cells normalized to 0.1% DMSO data are presented for INSTIs tested in cell viability (Cell-Titer Glo) assay 48 hours after transfection. BIC (n=5), CAB (n=3), DTG (n=2), EVG (n=3), RAL (n=3), and p8 (n=3) show minimal effects on cellular toxicity across concentrations tested. An ordinary one-way ANOVA with Dunnett's multiple comparisons test was used to compare % GFP levels to the DMSO control, $*P \leq 0.05$, $**P \leq 0.01$, $***P \leq 0.001$, unmarked datapoints = not significant.
